# Assessing inclusion of trans people in HIV national strategic plans: a review of 60 high HIV prevalence countries

**DOI:** 10.1002/jia2.25837

**Published:** 2021-11-11

**Authors:** Jennifer Sherwood, Elise Lankiewicz, Erika Castellanos, Naomhán O'Connor, Liesl Theron, Arjee Restar

**Affiliations:** ^1^ Public Policy Office amfAR Foundation for AIDS Research Washington DC USA; ^2^ GATE New York USA; ^3^ Independent Consultant Johannesburg South Africa; ^4^ Department of Epidemiology Johns Hopkins Bloomberg School of Public Health Baltimore Maryland USA

**Keywords:** HIV, national strategic plan, transgender, trans, key populations, strategic planning

## Abstract

**Introduction:**

Trans people are disproportionately impacted by HIV yet have not been adequately prioritized in national HIV responses or policy documents. This review aims to understand the extent of meaningful inclusion of trans people in national strategic plans (NSPs) for HIV/AIDS as an essential step in ensuring that HIV policy aligns with epidemiologic data, and trans‐specific programming is funded, implemented and sustained.

**Methods:**

HIV NSPs from 60 countries, across five global regions, were assessed for the level of inclusion of trans populations between January and March 2021. The most recently available NSP for each country, published after 2011, was obtained through publicly accessible online sources or through researcher networks. Data were manually extracted from NSPs using a framework of indicators focusing on trans inclusion in these five major sections of NSPs: (1) narratives; (2) epidemiological data; (3) monitoring and evaluation (M&E) indicators and targets; (4) activities; and (5) budgets.

**Results and discussion:**

Within all reviewed NSPs, 65.0% (39/60) mentioned trans people in at least one of the five key sections but only 8.3% (5/60) included trans people in all five key sections. Trans people were more commonly mentioned in the background/narratives of NSPs (61.7%, 37/60) but less commonly included NSP activities (38.3%, 23/60), in M&E indicators and targets (23.7%, 14/60), in epidemiological data (20.0% 12/60), and in NSP budgets (13.3%, 8/60). Countries in the Asia and Pacific region most frequently included trans people in all five key sections (38%, 5/13), while no countries in Eastern and Southern Africa included trans people in all NSP sections.

**Conclusions:**

This analysis finds substantial gaps in the inclusion of trans populations in NSPs globally. Results highlight the pressing need for states, technical partners, and international funders to engage with trans communities to improve trans‐inclusion in all key sections of NSPs. Trans inclusion in NSPs is an essential step towards reaching the populations most at risk of HIV and ultimately achieving country‐level epidemic control.

## 1 INTRODUCTION

To achieve pandemic control, the global HIV response must reach key populations – the groups most vulnerable to HIV who are not provided adequate access to HIV services or equal legal protections under the law [1]. Key populations include trans people, who are at especially high risk of HIV and face widespread stigma and state sanctioned discrimination [2]. In this paper, trans is used as an umbrella term to include trans masculine or trans people assigned female at birth, trans feminine or trans people assigned male at birth, transgender people, non‐binary people, culturally specific gender identities that fall outside the male/female binary and all gender identities in which a person does not identify with their gender assigned at birth. An estimated 19% of trans women globally are living with HIV [2], compared to 0.7% of other adults of reproductive age [3]. HIV prevalence is even higher among trans sex workers, estimated to be 27.3% globally [4]. HIV surveillance data on trans men are limited. However, the social and structural drivers of HIV such as discrimination, gender‐based violence, and other human rights abuses are known experiences of trans people and likely contributors to heightened HIV risk in trans men [5]. Despite this disproportionate burden, trans populations have not been adequately prioritized in the HIV response and trans‐specific HIV programming continues to be underfunded [6, 7].

States play a crucial role in shaping trans inclusion in the country HIV response and funding allocations. Specifically, a country's national strategic plan (NSP) for HIV/AIDS is an important document identifying which populations and strategies are to be prioritized in the HIV response. Meaningful trans inclusion in NSPs is essential to ensuring that HIV policy aligns with epidemiologic data, and trans‐specific programming is funded, implemented and sustained. Additionally, international funders, including the Global Fund to Fight AIDS Tuberculosis and Malaria (GF) and the President's Emergency Plan for AIDS Relief (PEPFAR), may use NSPs to guide investments. Recent estimates showed that while trans people made up about 1% of new HIV infections in 2018, programs including trans people received only 0.06% of total global HIV funding [6]. Even within funding that is ostensibly trans‐targeted, the majority of these funds were funnelled through general population HIV programs. General population programs do not universally serve the needs of trans people, with some programs deemed inaccessible and ineffective in reaching trans populations [7]. Ensuring trans inclusive NSPs is vital to help direct international funding to the highest risk populations.

Previous review of NSPs has shown limited inclusion of trans populations in these key policy documents [8]. In a 2018 World Health Organization (WHO) review of NSPs in the Africa region, only 22% of the 45 NSPs reviewed mentioned trans people. This was the lowest level of inclusion of any key population group in the review [8]. Where trans people were mentioned, references were primarily within general discussions of key populations [8]. The review also noted that trans people were often inappropriately grouped in with men who have sex with men (MSM) [8], despite differing programmatic needs for this population. Beyond the WHO review, research to understand trans inclusion in NSPs has been limited. Specifically, there is a gap in understanding the extent of trans inclusion in NSPs from regions outside of Africa and little systematic work has been done to understand the quality of inclusion where it is occurring.

This analysis reviews HIV NSPs from five global regions for meaningful inclusion of trans populations in NSP key sections. This review purposefully highlights whether trans populations are included in targeting and budgeting sections of NSPs, which represent the most concrete government pledges for trans programming and funding in these policy documents. Results provide a more in‐depth understanding of the global and regional state of trans inclusion in HIV strategic planning and inform where country‐level gaps need to be addressed.

## 2 METHODS

Data for this review were collected between January and March 2021. Formal submission to an ethical review board was not required for this study, which includes only secondary data analysis of publicly available content not involving human subjects.

### 2.1 NSP selection and compilation

This review sought to include NSPs, published in or after 2011, from the countries most impacted by HIV in the five UNAIDS regions with the highest adult HIV prevalence [3]. Specifically, the 15 highest HIV prevalence countries were included from both the Eastern and Southern Africa region and the Western and Central Africa region [3]. The 10 most prevalent countries were included from Latin America and the Caribbean region, Eastern Europe and Central Asia region, and Asia and the Pacific region [3]. Additionally, any country that was a member of the Global HIV Prevention Coalition [9], but that was not already included in the review based on HIV prevalence (*n* = 7) was added. Global HIV Prevention Coalition countries were included given the relevance of this review to the Coalition's work on key populations inclusiveness policy. This inclusion criterion resulted in 67 countries in total included in the review. NSPs were originally searched for online, focusing on Ministry of Health websites for each country and other policy document databases, including the International Labour Organization, the AIDS Data Hub and the HIV Health Clearinghouse [10, 11, 12]. Where authors were unable to locate NSPs through these methods, regional UNAIDS and WHO offices were contacted to request NSPs.

### 2.2 NSP data extraction

A framework for data extraction was created with five core indicators, and 19 sub‐indicators, focusing on trans inclusion in the major sections of NSPs (Table 1). Indicators measure to what extent trans populations are included in: (1) NSP narrative sections; (2) epidemiological data; (3) monitoring and evaluation (M&E) indicators and targets; (4) NSP activities; and (5) NSP budgets. M&E indicators and targets were originally reviewed separately, but the results were nearly identical so are reported together throughout. All indicators were co‐designed with trans‐led global NGO *GATE* [13] and refined through discussions between researchers and community members. Only the NSP document itself and linked annexes or appendices were considered in the review. Separate evaluation frameworks or country documents outside of the NSP were not included.

**Table 1:** List of indicators

Indicators	Possible responses
Are trans people mentioned in the narrative section of the plan in any way? Are trans people referenced generally as transgender/trans? Are trans people referenced specifically as trans women? Are trans people referenced specifically as trans men? Are trans people referenced specifically as trans sex workers?	Yes No
2. Does the plan include any country‐specific trans epidemiological data in the narrative sections? Does the plan include country‐specific trans prevalence or incidence estimates? Does the plan include country‐specific trans size estimates?	Yes No
3. Are there trans‐specific monitoring and evaluation (M&E) indicators/targets? Are there trans‐specific indicators/targets for general epidemiology? Are there trans‐specific indicators/targets for prevention? Are there trans‐specific indicators/targets for testing or linkage? Are there trans‐specific indicators/targets for treatment? Are there trans‐specific indicators/targets for retention or viral suppression?	Yes, listed separately from other KP groups Yes, listed with other KP groups No
4. Are there trans‐specific activities? Are there trans‐specific activities for prevention? Are there trans‐specific activities for testing or linkage? Are there trans‐specific activities for treatment? Are there trans‐specific activities for retention or viral suppression?	Yes, listed separately from other KP groups Yes, listed with other KP groups No
5. Does the plan budget for trans‐specific activities? Does the plan budget for trans‐specific prevention activities? Does the plan budget for trans‐specific testing or linkage activities? Does the plan budget for trans‐specific treatment activities? Does the plan budget for trans‐specific retention or viral suppression activities?	Yes, listed separately from other KP groups Yes, listed with other KP groups No

All NSPs were reviewed against the indicator list and data were extracted by one study investigator (EL). Where it was not clear whether an NSP met an indicator criterion, initial extraction was reviewed by a second investigator (JS) and any discrepancies were discussed to yield agreement. NSPs written in languages other than English were translated using Google Document Translation [14]. For two countries, Indonesia and Belarus, where document formatting did not allow for Google translation, fluent speakers from researchers’ partner organizations were trained on analysis methodology and performed the data extraction.

Data extraction was designed to be as inclusive as possible to find any mention or reference to trans populations in the NSPs. All known terms for “trans,” or people whose gender identity or expression differs from their sex assigned at birth, such as “hijra,” “waria,” “apwint,” “transgender” or “transsexual,” were considered to be equivalent to trans for data extraction purposes. Additionally, the conditions of any indicator only had to be met once at any point in the NSP to be marked affirmatively. All M&E indicators/targets, including process/output, outcome or impact, were included in the review.

### 2.3 Indicator coding

For indicators related to NSP narratives and epidemiological data, possible responses were either “yes,” trans people were included at least once, or “no,” there was no mention of trans people by name in narrative or data sections of the NSP. For indicators related to NSP M&E indicators/targets, activities or budgets, there were three possible responses: (1) “no, there was no inclusion of trans people”; (2) “yes, there was mention of trans people in a group with other key populations”; or (3) “yes, there was mention of trans people and they were mentioned separately from other key populations groups.” This distinction between affirmative response options #2 and #3 was made given the historical grouping of trans populations with other key populations in HIV data and programming [15, 16]. In the current review, if trans people were defined as a key population in the NSP narrative, but then targets, indicators, activities or budgets only referenced key populations generally – rather than specifically mentioning trans populations – this was not counted affirmatively as trans‐specific programming.

## 3 RESULTS AND DISCUSSION

### 3.1 NSP compilation results

The initial electronic search for the 67 country NSPs yielded 50 NSPs. Regional offices provided an additional 10 NSPs. NSPs published in the past 10 years (2011–2021) could not be located for seven of the 67 countries in the sampling frame, including Azerbaijan, Brazil, Equatorial Guinea, Kazakhstan, Mexico, Singapore and Uzbekistan. Data were extracted from the remaining 60 NSPs, applying to the years 2011–2030 [17, 18, 19, 20, 21, 22, 23, 24, 25, 26, 27, 28, 29, 30, 31, 32, 33, 34, 35, 36, 37, 38, 39, 40, 41, 42, 43, 44, 45, 46, 47, 48, 49, 50, 51, 52, 53, 54, 55, 56, 57, 58, 59, 60, 61, 62, 63, 64, 65, 66, 67, 68, 69, 70, 71, 72, 73, 74, 75, 76].

### 3.2 Regional summary results

Among all included NSPs, 65.0% (39/60) mentioned trans people in at least one of the five core sections of their NSP and 8.3% (5/60) of countries included trans people in all five key sections. The review showed 61.7% (37/60) of NSPs mentioned trans people within the narrative section of the plan in some way (Table 2). Narrative inclusion was highest regionally in Latin America and the Caribbean where 88.9% (8/9) of NSPs mentioned trans people. Narrative inclusion was lowest in Eastern Europe and Central Asia where only 28.6% (2/7) of NSPs reviewed mentioned trans people. Country‐specific epidemiologic data for trans people were included in 20.0% (12/60) of all NSPs. NSPs in Asia and the Pacific most frequently included trans epidemiological data (69.2%, 9/13), while no countries in Eastern and Southern Africa (0/16) or Eastern Europe and Central Asia (0/7) included this information.

**Table 2:** Percent of NSPs (2011–2030) that include trans people in five key sections

	Narrative	Country and trans‐specific epidemiological data	Trans‐specific indicators/targets	Trans‐specific activities	Budgeting for trans‐specific activities
Region	% (*n*)	% (*n*)	% (*n*)	% (*n*)	% (*n*)
Asia and the Pacific (*n* = 13)	84.6 (11)	69.2 (9)	46.2 (6)	84.6 (11)	38.5 (5)
Eastern and Southern Africa (*n* = 16)	62.5 (10)	0.0 (0)	18.8 (3)	18.8 (3)	0.0 (0)
Eastern Europe and Central Asia (*n* = 7)	28.6 (2)	0.0 (0)	14.3 (1)	42.9 (3)	14.3 (1)
Latin America and the Caribbean (*n* = 9)	88.9 (8)	22.2 (2)	22.2 (2)	44.4 (4)	11.1 (1)
Western and Central Africa (*n* = 15)	40.0 (6)	6.7 (1)	13.3 (2)	13.3 (2)	6.7 (1)
Total (*n* = 60)	61.7 (37)	20.0 (12)	23.3 (14)	38.3 (23)	13.3 (8)

*Note*: The values in this table come from the five core indicators (Table 1).

Across all NSPs reviewed, 23.3% (14/60) included indicators/targets specific to trans people. Trans‐specific indicators/targets were more common in NSPs from Asia and the Pacific (46.2%, 6/13) and were least common in Western and Central Africa (13.3%, 2/15). Trans‐specific activities were found in 38.3% (23/60) of all NSPs. Trans‐specific activities were most common in NSPs from Asia and the Pacific (84.6%, 11/13) and least common in NSPs from Western and Central Africa (13.3%, 2/15). Finally, few NSPs (13.3%, 8/60) included mention of trans people in their budgets. Regionally, Asia and the Pacific had the highest level of trans inclusion in NSP budgets (38.5%, 5/13) and Eastern and Southern Africa did not have any NSP budgets that included trans people (0.0%, 0/16).

### 3.3 Narratives

**Figure 1:**
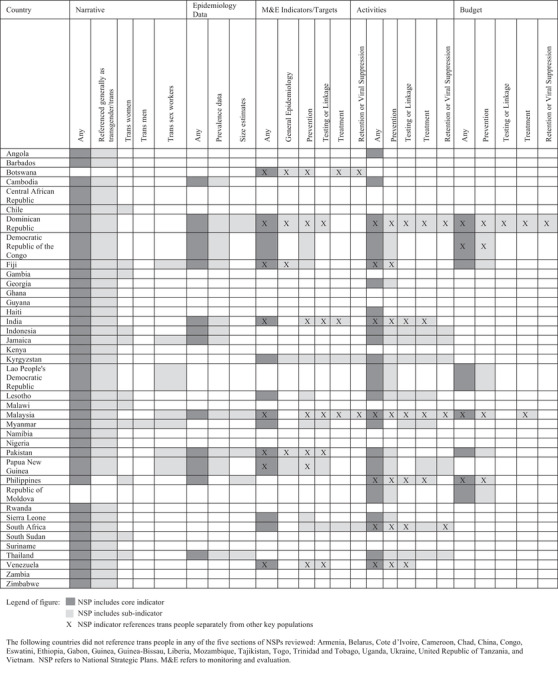
Trans‐specific inclusion in NSP narratives, epidemiology, M&E indicators/targets, activities and budgets

Within the narrative section of NSPs, 60.0% (36/60) referenced trans populations generally (Figure 1). Fewer countries mentioned, specifically, trans women (18.3%, 11/60), trans sex workers (11.7%, 7/60) and trans men (1.7%, 1/60). Some countries only referred to trans people within umbrella initialisms like “LGBTI” (Haiti [42]) or “LGBT” (Kenya [46]). Only one country, Myanma

r, made brief reference to trans men [54]. A quarter (25.0%, 15/60) of the NSPs in this study referenced trans people in the narrative but not in any other section of the NSP that was reviewed (epidemiological data, indicators/targets, activities or budgets). Narrative inclusion of trans people in NSPs was often in the context of defining or describing key or vulnerable populations in a country (Table 3).

**Table 3:** Affirmative examples of trans inclusion in NSPs

*Narrative inclusion: Are trans people mentioned in the narrative section of the plan in any way?*	*Yes: “*Gender inequalities between and among categories of men and women– or those who assume feminine and masculine behaviours and roles such as transgender persons – are an underlying determinant of HIV risk and vulnerability in Jamaica." Jamaica [45] p. 35
*Epidemiological data inclusion: Does the plan include any country‐specific trans epidemiological data in the narrative sections?*	*Yes*: “Key Population estimations were revised during the 2014 Spectrum exercise in collaboration with UNAIDS Asia Pacific Regional Support Team…Hijra/transgendered Sex Workers (HSW): 50, 598." Pakistan [57] p. 17
*Indicator/target inclusion: Are there trans‐specific M&E indicators/targets?*	*Yes, listed with other KP groups*: “Knowledge of HIV status among key populations (by population) MSM/TG Target 2022: 85%.” Papua New Guinea [58] p. 22
*Activity inclusion: Are there trans‐specific activities?*	*Yes, listed separately*: Within the section for Mitigating Sexual Transmission among Transgender, “Community led HIV/STI services.” Malaysia [52] p. 83
*Does the plan budget for trans‐specific activities?*	*Yes, listed with other KP groups*: The budget includes peer education and condoms and notes high‐risk MSM/TG people will be targeted. Lao People's Democratic Republic [48] p. 47

Abbreviations: KP, key populations; M&E, monitoring and evaluation; MSM, men who have sex with men; NSP, National Strategic Plans; TG, trans people.

### 3.4 Epidemiological data

Overall, 20% (12/60) of countries included trans‐specific epidemiological data. The most common trans‐specific data included were HIV prevalence data (18.3%, 11/60). Less commonly, countries provided trans population size estimates (8.3%, 5/60) (Figure 1). Countries that included trans people in estimates of MSM but had no separate estimates, such as Lao People's Democratic Republic [48], were not considered to be trans‐specific. In several countries, including Fiji [34], Papua New Guinea [58] and Pakistan [57], at least one of the size or prevalence estimates were specific to the trans sex worker population (Table 3). Of note, some of the epidemiological data used in current NSPs were a decade old – for example, the Papua New Guinea NSP that covers 2018–2022 relies on trans sex worker HIV prevalence data from 2011 [58].

### 3.5 Indicators/targets

All NSPs with any trans‐specific indicators/targets (23.7%, 14/60) set a trans‐specific indicator/target for HIV prevention (e.g. for improving condom use [30]). Trans‐specific indicators/targets were less common further along the HIV care continuum with 15.0% (9/60) of NSPs having a trans‐specific testing or linkage indicator/target, 8.3% (5/60) having a trans‐specific treatment indicator/target and 6.7% (4/60) having a trans‐specific retention or viral suppression indicator/target. Three countries, Malaysia [52], Kyrgyzstan [47] and South Africa [63], included trans‐specific targets for every category of the HIV continuum of care. Only 10.0% (6/60) of NSPs included a trans‐specific general epidemiology indicator/target. Of the 14 NSPs with trans‐specific indicators/targets, the majority (57.1%, 8/14) listed trans people separately from other key populations groups in at least one indicator/target. Some NSPs grouped targets for trans people and MSM together (Table 3).

### 3.6 Activities

More NSPs (38.3%, 23/60) included trans‐specific activities as compared to trans‐specific epidemiological data, indicators/targets or budgets (Figure 1). Along the HIV continuum of care, 31.7% (19/60) of NSPs had trans‐specific prevention activities, 20.0% (12/60) had activities for testing/linkage, 20.0% had activities for treatment (12/60) and 13.3% (8/60) had retention/viral suppression activities. As was observed for trans indicator/target data, trans‐specific activities were less common further along the HIV care continuum. Seven countries (11.7%) included trans‐specific activities across the entirety of the HIV care continuum (Dominican Republic [30], Jamaica [45], Kyrgyzstan [47], Malaysia [52], South Africa [63], Thailand [67] and Indonesia [44]). About a third of the NSPs with activities specific to trans people (7/23) listed trans people separately from other key population groups. Some NSPs specifically mentioned that the community would be responsible for implementation of trans‐specific activities (Table 3). Of note, the sub‐indicator categories we included did not capture all trans‐specific activities. Activities that specifically included trans people but fell outside of the categories included in our sub‐indicators were: conducting seroprevalence studies among key population groups, including trans people (Angola [17]); creating a more inclusive legal and policy framework to support trans people (Cambodia [23]); and capacity building for community organizations to address stigma against trans people (Haiti [42]). These countries are noted as having “any” trans‐specific activities in Figure 1.

### 3.7 Budgets

Of the 13.3% (8/60) of NSPs, including trans people anywhere in their budget, all countries budgeted for trans‐specific prevention activities (e.g. condom and lubricant procurement [59]) (Figure 1). Two countries (3.3%), the Dominican Republic and Malaysia, also budgeted for trans‐specific treatment activities (e.g. treatment support [30]). Only the Dominican Republic also included mention of trans people in budgeting for testing/linkage or retention activities/viral suppression (1.7%, 1/60). While some NSPs specified that activities should be led by trans people or organizations, none specified that funding would go to trans‐led organizations to implement these activities. However, the vast majority of NSP budget sections did not provide information on what organizations would receive funding to implement any activities in general. Half of the NSPs that included any trans‐related budget items listed trans people separately from other key population groups (50%, 4/8).

## 4 DISCUSSION

The results of this analysis demonstrate lack of consistent and substantive inclusion of trans people in HIV NSPs globally. Only five of 60 countries in this review included trans people in all key sections of their NSP and over a third of NSPs did not mention trans people anywhere in the document. Given the disproportionate impact of the epidemic on trans people, the inclusion of this population in national and global strategic planning documents is critical to ensuring the implementation of quality trans‐specific programming and ultimately achieving epidemic control. States and international funders must work to include trans‐specific approaches throughout the entirety of NSPs, addressing all stages of the continuum of care with the input of trans communities.

Overall, NSPs from the regions of Asia and the Pacific, followed by Latin America and the Caribbean, had more substantive inclusion of trans people as compared to Eastern and Southern Africa, Eastern Europe and Central Asia, and Western and Central Africa. This may be a direct result of the strong trans organizing, successful advocacy, community‐generated data and ongoing work towards legal gender recognition in these regions. Geographical differences in trans inclusion in NSPs call for region‐specific or country‐specific responses to increase trans inclusion in NSPs. For example, in some countries where trans people are left out of NSPs completely, advocates may need to focus on increasing state recognition of trans people or securing national research studies among trans populations. In other countries where there is already significant inclusion of trans people in NSPs, advocates could focus on improving the quality of that inclusion. This might include increasing trans‐specific HIV targets or funding for trans programming across the continuum of care.

In addition, our review showed that, where trans inclusion did occur, it was most often in the narrative or activities sections of NSPs, rather than the M&E and budgeting sections. Trans inclusion in only the narrative section of NSPs creates little in the way of state accountability for implementing trans programming. These findings align with those of a previous review of NSPs in the WHO Africa Region, which found inclusion of transgender people in NSPs occurred most often in NSP narrative sections describing key populations [8]. While this is a necessary start, trans inclusion in NSP narratives does not ensure that actual programming will reach trans communities. To effectively control national HIV epidemics, there is an urgent need for policymakers to ensure that trans people are included across all aspects of NSPs, including in the targeting and budget sections. There is an additional requirement for monitoring and oversight to ensure that plans are implemented accordingly.

Quality trans inclusion in NSPs often relies on the availability of population size estimates and country‐specific HIV prevalence estimates for the trans community. The majority (80%) of NSPs reviewed in this study did not include trans‐specific HIV prevalence or size estimate, likely because these data are unavailable [77]. A previous review of key population size estimates in the Africa region found that there were no studies offering country‐level trans population size estimates [77]. Currently, many health surveillance systems are not designed to include data around trans people [78, 79], an omission that has created substantial gaps in global health data for these populations. Lack of epidemiological data for trans people can create challenges to promoting resource allocation for trans‐specific programming, especially in the context of generalized HIV epidemics with many competing priorities. However, where HIV prevalence data for trans people do exist, prevalence estimates often far exceed those of the general population [2]. It is reasonable to assume that even in countries without HIV data collection efforts among trans people, that trans populations are still at increased risk. Indeed, for key populations in general, existing data show that key populations bear a disproportionate HIV prevalence even in generalized epidemics [80]. To inform trans programming and resource allocation, it is vital that investments in trans population size estimate studies are increased and that trans people are included in HIV epidemiological surveillance surveys.

Additionally, this analysis shows that where NSPs do include trans people, they are frequently only referenced collectively with other key populations. This lack of specificity makes it difficult to discern if trans‐specific services are being planned and provided within the larger umbrella of “key population” services. Trans people and other key populations have unique structural, behavioural and biological risk factors, making tailored population‐specific programming critical [80]. Even where combined key population interventions are appropriate, disaggregated monitoring and evaluation is essential to ensuring programming is reaching all intended populations. Many NSPs in this review grouped trans women with MSM in their description of populations, epidemiological data and indicators/targets. The ongoing subsummation of trans women into the MSM population erases the specific HIV‐related experiences and vulnerabilities of trans women and results in less effective HIV programming [15, 81]. For example, HIV programming designed for MSM will likely not meet the needs of trans women, who need HIV services consistent with their gender identity. Successful HIV programming for trans populations will offer gender affirming health services, in addition to welcoming HIV services, making it comfortable and desirable for trans people to access HIV services [82].

Overall, this analysis demonstrates the need for improved inclusion of trans people in HIV NSPs globally. Specifically, NSPs must incorporate trans people in all key sections and include budgets for trans‐specific programming. Trans programming should be trans‐led and must explicitly address gender identity inequities given the social stigma and structural discrimination faced by trans communities [83]. To achieve appropriate inclusion, states should invest in processes to engage in‐country trans advocates and organizations in NSP design and evaluation. This is aligned with the Greater Involvement of People Living with HIV (GIPA) principle, which describes the right of people living with HIV to participate in the decision‐making processes that impact their lives [84]. Given the challenges many trans organizations face in engaging with state‐led processes, including even registering as a trans‐led organization, states should be proactive and intentional in facilitating this engagement. Adequate trans inclusion in the processes to develop NSPs should be assessed based on trans advocates assessment of whether or not opportunities for engagement are substantive.

International donors, such as the Global Fund, PEPFAR, and UNAIDS, should also provide technical assistance and direct funding to strengthen trans community organizations. This support is vital for communities to develop and implement advocacy plans with national governments to improve NSPs and generate data. In addition, donors and technical partners should work with states and the trans community to improve the specificity of NSPs, with a focus on clear separation of key populations in each section. Donors can include requirements for trans inclusion where they fund size estimate or behavioural studies for use in NSPs. Detailed population‐specific approaches in NSPs are vital to accurately evaluating the extent of engagement and national investment in key populations. International donors can also use funding to encourage states to improve trans inclusion in NSPs through contractual requirements and programmatic guidance. This role is particularly critical given that these donors hold significant sway in engaging states even where trans civil society organizations face stigmatization or criminalization that makes advocacy efforts particularly challenging.

### 4.1 Limitations

There were several limitations to this review. We were not able to locate several NSPs and a number of NSPs reviewed were not current. It is not known whether more recent NSPs existed that we were unable to obtain, or if new NSPs had not yet been produced. At least two NSPs, Guinea‐Bissau [40] and South Sudan [64], were provided to us in draft version, so the data extracted may not exactly reflect the final version. Except for NSPs from Indonesia [44] and Belarus [20], all non‐English NSPs were translated using Google Translate, which at times may have resulted in loss of clarity or nuance during data extraction. NSPs differ in level of detail, meaning that where we noted lack of trans inclusion, this may indicate actual exclusion of trans people in a country's planning, and/or it may be reflective of an undetailed NSP. In either scenario, the results of this review point to the need to improve NSPs, both through inclusion of trans populations and improving the technical quality of the documents. Additionally, the commitments made in NSPs, including for trans programming and funding, are not always actualized. As a result, this review is only able to provide information on inclusion of trans populations in NSP documents and not on the extent to which trans programming is being implemented or services are reaching trans communities.

## 5 CONCLUSIONS

This analysis reveals a significant gap in the meaningful inclusion of trans people in NSPs. There is a pressing need for states and international funders to engage with trans communities to understand how best to meet their needs and to incorporate this information into strategic planning documents. Trans inclusion in NSPs should occur across all key sections of the document, be tailored to the trans experience and be disaggregated from other key population groups. Specific attention to trans communities in NSP documents should be seen as an essential step in the process of creating and implementing high‐quality HIV programming necessary for country‐level epidemic control.

## COMPETING INTERESTS

The authors have no competing interests to declare.

## AUTHORS’ CONTRIBUTIONS

AR and JS conceptualized the paper. EL and JS were responsible for data extraction and analysis. EL and JS drafted the paper. AR, EC, LT and NO provided significant edits to the paper. All authors have read and approved the final manuscript.

## FUNDING

This study was funded by the Elton John AIDS Foundation.

## DISCLAIMER

The content is solely the responsibility of the authors and does not necessarily represent the official views of the agencies, institutions or organizations listed.

## Data Availability

All National Strategic Plans and data extracted for this analysis are available from the authors upon request.
